# Distributions and Sources of Polycyclic Aromatic Hydrocarbons (PAHs) in Soils around a Chemical Plant in Shanxi, China

**DOI:** 10.3390/ijerph14101198

**Published:** 2017-10-09

**Authors:** Haihua Jiao, Qi Wang, Nana Zhao, Bo Jin, Xuliang Zhuang, Zhihui Bai

**Affiliations:** 1 Department of Biological Sciences and Technology, Changzhi University, Changzhi 046011, China; jiaohaihua68@163.com (H.J.); wangqiwq@126.com (Q.W.); znn1140655116@163.com (N.Z.); 2 Research Center for Eco-Environmental Sciences, Chinese Academy of Sciences, Beijing 100085, China; xlzhuang@rcees.ac.cn; 3 School of Chemical Engineering, The University of Adelaide, Adelaide, SA 5005, Australia; bo.jin@adelaide.edu.au; 4 College of Environment & Resources, University of Chinese Academy of Sciences, Beijing 100049, China

**Keywords:** polycyclic aromatic hydrocarbons (PAHs), soil contamination, chemical plant, organic pollutants

## Abstract

*Background*: Yearly the Shanxi coal chemical industry extracts many coal resources, producing at the same time many polycyclic aromatic hydrocarbons (PAHs) that are emitted as by-products of coal incomplete combustion. *Methods*: Sixty-six soil samples collected from 0 to 100 cm vertical sections of three different agricultural (AS), roadside (RS) and park (PS) functional soils around a chemical plant in Shanxi, China were analyzed for the presence of the 16 priority control PAHs. *Results*: The total concentrations (∑16PAHs) varied in a range of 35.4–116 mg/kg, 5.93–66.5 mg/kg and 3.87–76.0 mg/kg for the RS, PS and AS surface soil, respectively, and 5-ring PAHs were found to be dominant (44.4–49.0%), followed by 4-ring PAHs (15.9–24.5%). Moreover, the average value of ∑16PAHs decreased with the depth, 7.87 mg/kg (0–25 cm), 4.29 mg/kg (25–50 cm), 3.00 mg/kg (50–75 cm), 2.64 mg/kg (75–100 cm) respectively, in PS and AS soil vertical sections. *Conclusions*: The PAH levels in the studied soils were the serious contamination level (over 1.00 mg/kg) according to the Soils Quality Guidelines. The carcinogenic PAHs (ΣBPAHsBapeq) were approximately 14.8 times higher than the standard guideline level (0.60 mg/kg) and 90.3% of PAHs were produced by coal/wood/grass combustion processes.

## 1. Introduction

Polycyclic aromatic hydrocarbons (PAHs) are a complex group of chemicals containing two or more aromatic rings. The PAHs are products of incomplete combustion, present in petroleum products, and as such are commonly encountered as contaminants at hazardous waste sites. PAHs occur in the environment as complex mixtures of many components with widely varying toxic potencies. Some PAHs are carcinogens and mutagens. Sixteen PAHs have been listed as priority pollutants by the United States Environmental Protection Agency [1] and eight of them have been classified as probable or possible human carcinogens by the International Agency for Research on Cancer [2,3].

PAHs are derived mostly from anthropogenic sources such as combustion of coal, fuel and grass [4]. Coal is a complex heterogeneous mixture of organic and inorganic constituents of allothigenic or authigenic origin [5]. It is very useful in power generation, domestic and industrial heating and in the synthesis of chemicals such as gasoline, ammonia and urea, etc. Many deleterious compounds are found in coal which may be released into the environment during coal mining combustion, coking, pyrolysis, and other coal preparation processes to make the coal consistent in quality and suitable for selling [6]. The PAHs are the main toxic pollutants emitted from chemical plants using coal [7], as large amounts of PAHs are formed and emitted as by-products of the incomplete combustion of coal. Consequently, communities surrounding chemical plants can be placed at increased risk of adverse health outcomes [8].

Many studies on the sources and distributions of PAHs in surface soils have been reported in the last decade [9,10,11,12]. The molecular marker approach has been widely used to identify the sources of hydrocarbon compounds [13,14]. However, most studies have been focused on PAH pollution in urban, mine and oil field soils [15,16,17], but seldom in chemical plant areas. PAH soil pollution near chemical plants could cause public health risks since residences and parks are nearby [18,19]. In this study, we investigated the PAH distribution and characteristics in terms of concentrations and compositions in contaminated soil at a vertical depth of 0–100 cm, and an area of 0–1500 m around a chemical plant located in Changzhi district (Shanxi, China). Samples were analyzed and characterized by PAH ratios and composition. The aim of this study was to provide baseline information on the levels of PAH contamination in the vicinity of the chemical plant. The data can be useful for assessment of the environmental impact of PAHs associated with chemical plants.

## 2. Materials and Methods

### 2.1. Sample Collection

**Figure 1:**
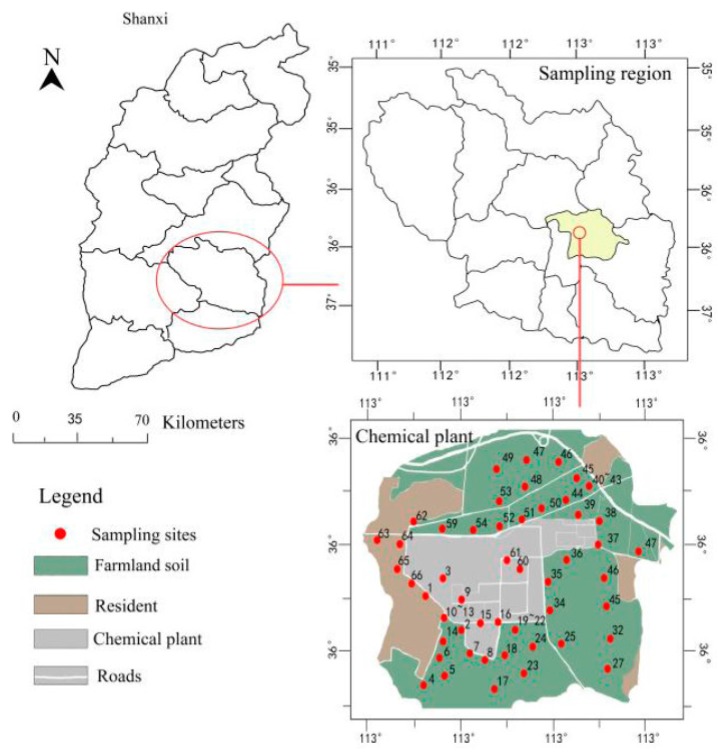
A map showing the sampling area and sites.

To get a better understanding of PAH distribution and characteristics with respect to the PAH sources, we designed representative soil sampling sites based on the PAHs released from coal combustion from a chemical plant and agriculture regions and transportation areas. Sampling was conducted at these sites in September 2016. Sixty-six samples were randomly collected for the determination of the PAH distribution in the soil from the areas around the chemical plant. Fourteen samples were collected from agricultural soils (AS), twenty seven samples were collected from roadside soils (RS), and ten from park green space soils (PS) around the chemical plant. Agricultural soil refers to the soil that is used for agricultural production. Roadside soil refers to soil along roads and road junctions. Park green space soil refers to soil from gardens, greenbelts, and so on. On the average, one sampling point represented about 500–600 m^2^ (30–40 point soils then combined together as one sample), Global Positioning System (GPS) data was used to identify the sampling positions (Figure 1). Fifty one samples were collected from surface (0–25 cm) soil around the chemical plant. Fifteen PS and AS soil samples were collected from different depths (25–50 cm, 50–75 cm, 75–100 cm layer). These types of soils are affected by the chemical plant activities. The sampling sites were selected in order to reflect the diverse exposure of soils and duration of exposure to the pollution sources. They represent samples directly affected by industrial emissions, and directly subjected to vehicles emissions. The soil samples obtained were transported to the Analytical Laboratory at Changzhi University, China. The soil samples were air-dried at room temperature after removing stones and residual roots, and passed through a 1 mm sieve to remove the coarse soil fraction. Soil samples were stored at −20 °C before analysis.

### 2.2. Sample Extraction

According to the modified standard method [15], soil samples (10 g dry weight (dw)) and anhydrous sodium sulfate (5 g) were added into a 50 mL glass flask and mixed with 30 mL acetone: dichloromethane solvent (1:1 *v*/*v*). The sample was extracted for 15–20 min in an ultrasonic agitation apparatus (SB 5200 DT, Ningbo Xingzhi Biological Technology Co. Ltd., Ningbo, China), and the final extract was filtered through a column (15 cm length × 10 mm id) filled with 10 cm dried anhydrous sodium sulfate to remove the residual water, and the process was repeated twice again with another 30 mL of solvent. The final extracts were concentrated to around 2 mL by a gentle stream of nitrogen at 35–40 °C, and cleaned using a 0.2 µm organic phase membrane filter (Varian Inc., Lake Forest, CA, USA).

### 2.3. PAHs Analysis

PAHs analyses were carried out using a gas chromatography mass spectrometer (GC/MS) system (Shimadzu Enterprise Management (China) Co. Ltd., Shanghai, China) equipped with a GC-MS QP2010 Ultra column (30 m length × 0.25 mm id × 0.25 μm film thickness, HP-5 MS capillary column) using helium as the carrier gas. The GC-MS analysis was operated in the selected ion monitoring (SIM) mode, injection volume was 1 μL in split less mode. Temperature was first set up at 50 °C for 1 min, and then gradually increased to 200 °C at 20 °C/min held for 1 min, 260 °C at 6 °C/min held for 1 min, and 290 °C at 20 °C/min held for 10 min. The 16 individual PAHs (AccuStandard, New Haven, CT, USA), including 16 USEPA priority pollutants listed in Table 1 were analyzed in each sample.

### 2.4. Quality Control

Quantitation was performed using an external standard calibration method (five-point calibration; 0.01, 0.05, 0.10, 0.20 and 0.40 mg/L), and correlation coefficients (R^2^) for the calibration curves that were all greater than 0.9998. Analytical methods were checked for precision and accuracy. Limits of detection (LODs) were calculated based on the ratio of three times of the standard deviation of the response of seven replicate measurements and the slope of the calibration graph. LODs of PAHs varied in a range of 0.04 × 10^−3^–0.54 × 10^−3^ mg/kg dw. The repeatability of the method was assessed by analysis of six soil samples that had previously been extracted with the extraction mixture, and were supplemented at 0.10 mg/kg with a standard mixture (USEPA PAH standard mixture 2.00 mg/mL in methanol; Supelco, Bellefonte, PA, USA). Matrix spike experiment recoveries of certified reference materials ranged from (85 ± 3%, Acel) to (99 ± 6%, Phe) for the 16 PAHs. Blanks which contained no detectable PAHs were run periodically. The variation coefficients of PAHs concentration in duplicates were less than 15%.

### 2.5. Data Analysis

Data analysis was performed using means of the statistical package SPSS 17.0 software (SPSS Statistics Data Editor, Version 17.0, SPSS China, Beijing, China). Arc GIS 9.0 was used to draw the map of the sampling sites. All data was processed by Microsoft Excel (Microsoft Inc., Redmond, WA, USA) and diagrams were drawn using Origin 8.5 (Originlab Corporation, Wellesley Hills, MA, USA).

## 3. Results

### 3.1. Characteristics of PAHs Concentrations in Surface Soils

The 16 USEPA priority PAHs were quantified. The total concentration of the 16PAHs (∑16PAHs) in surface soils around the chemical plant are shown in Table 1. The values of ∑16PAHs varied in a range of 35.4–116 mg/kg (mean 65.5 mg/kg, median 60.7 mg/kg) that were close to the typical PAH concentrations in RS, and higher than typical concentrations in PS (range 3.82–36.4 mg/kg, mean 14.6 mg/kg, median 16.8 mg/kg) and AS (range 3.87–76.0 mg/kg, mean 12.6 mg/kg, median 7.55 mg/kg).

Moreover, the human carcinogen compounds (Bap, Daa, Baa, Bbf, Bkf, Chr, Ind and Nap) also were investigated in the surface soils of these sampling areas (AS, PS and RS), and results are presented in Table 1. The highest total carcinogenic PAHs (∑BPAHs) were distributed in RS with a range of 20.2–94.2 mg/kg (mean: 42.3 mg/kg), followed by PS (range: 3.82–36.4 mg/kg, mean: 14.6 mg/kg), and AS with the lowest (∑BPAHs) in a range of 3.11–66.1 mg/kg (mean: 11.2 mg/kg).

Bap is a typical PAH which is of greatest interest in terms of potential cancer hazard. Bap concentration varied in a range of 0.232–5.95 mg/kg, 0–35.3 mg/kg, 0.435–22.2 mg/kg for the AS, RS and PS, respectively (Table 1).

It is worthwhile to note that ∑16PAHsin these contaminated areas were 12.6–65.5 times higher than the standard level (1 mg/kg) of heavy pollution, while ∑BPAHs was 11.2–42.3 times higher than the standard level. Moreover, the concentrations of ∑16PAHs, ∑BPAHs and Bap were analyzed following an increasing order of AS < PS < RS. It could be assumed that the high PAHs are released from the chemical plant activity and heavy vehicular transportation, and due to the increasing of the distances away from the pollution sources, the highest level of PAHs in RS, but the lowest levels in AS.

An average concentration of 42.5 mg/kg of Σ16PAHs was analyzed in the soils around the chemical plant This average amount of Σ16PAHsis approximately 1.8–250 times higher than that those reported case studies for urban soils in Glasgow in the UK (11.9 mg/kg) and Ljubljana in Slovenia (0.989 mg/kg) [20], Bergen in Norway (6.78 mg/kg) [21], Tarragona in Spain (0.438 mg/kg) [22], and Hong Kong (0.17 mg/kg) [23], Beijing (3.92 mg/kg) [24], Tianjin (1.84 mg/kg) [25], Jinan (23.3 mg/kg) [26] and Dalian (6.44 mg/kg) in China [27]. The results indicate people should be cautious about the environmental quality of the area around the chemical plant.

### 3.2. Characteristics of PAHs Component in Surface Soils

**Figure 2:**
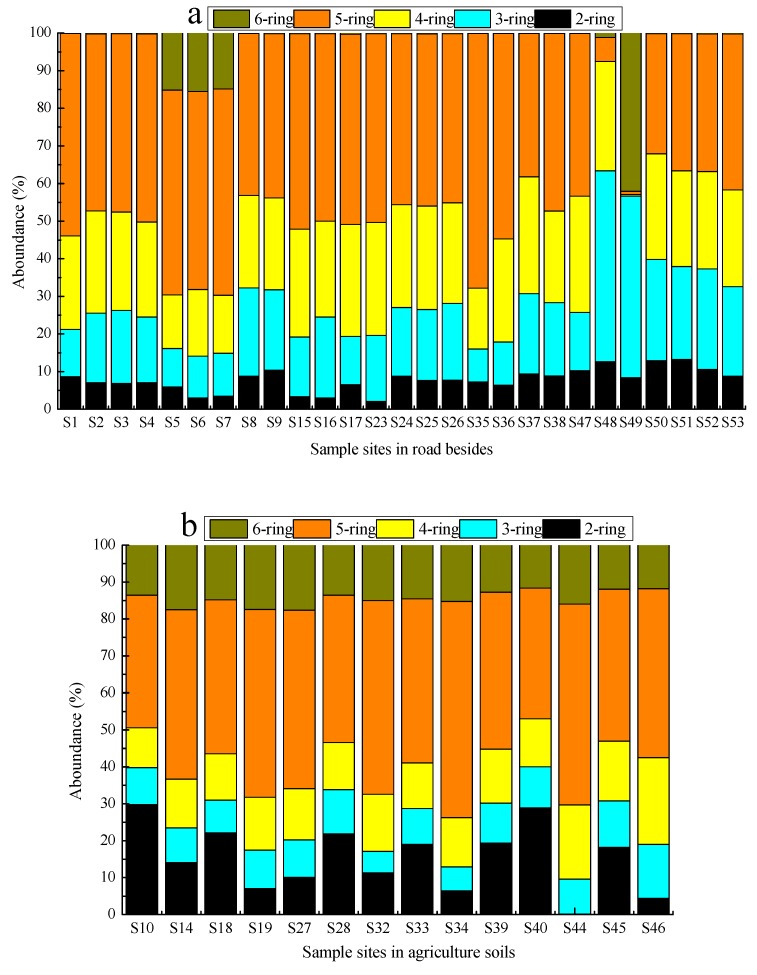

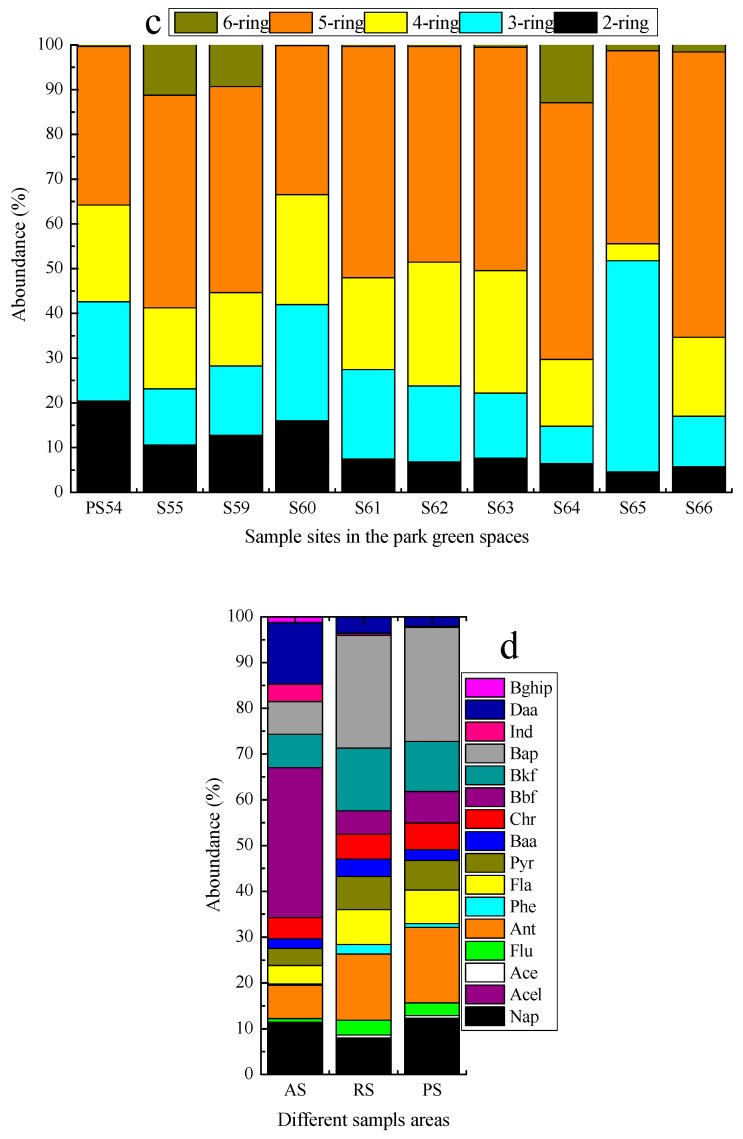
PAHs composition distribution in surface soils: (**a**) roadsides, (**b**) agriculture areas, (**c**) park green spaces, and (**d**) overall PAHcomponent distribution in all investigated samples. Notes: 2-ring (Nap); 3-ring (Ace, Acel, Flu, Phe, Ant); 4-ring (Fla, Pyr, Baa, Chr); 5-ring (Bbf, Bkf, Bap, Daa); 6-ring (Ind, Bghip).

PAHs represent complex chemicals which consist of multiple aromatic rings. According to the number of aromatic rings, the 16 PAHs are divided into five groups: 2-ring, 3-ring, 4-ring, 5-ring, and 6-ring PAHs. The distribution pattern of the 16 PAHs is shown in Figure 2. The sequence of the PAHs proportion in all surface soils was detected as 5-ring (34.9%)-4-ring (16.0%)-3-ring (13.4%)-2-ring (7.87%)-6-ring (5.05%). It is interesting to note that the soil samples in each area had different PAHs compositions in terms of the number of aromatic rings. 5-ring (44.1%) and 4-ring (24.5%) PAHs were analyzed as the most prominent compositions in the RS soils (Figure 2a). 5-ring (45.5%), 2-ring (15.2%) and 4-ring (14.7%) PAHs were found in AS (Figure 2b), while the PS samples were mainly consisted of 5-ring (47.6%) and 3-ring (19.5%) PAHs (Figure 2c). Therein, Bap, Bkf, Bbf, Ant, Nap and Ind were found to be the most prominent compounds in all soil samples. Bap was recorded with the highest ratio followed by Ant in RS soils.

Interestingly, these two compounds showed the same distribution pattern with higher ratio in PS, but had a significant different distribution pattern at AS, in which Bbf was determined with the highest ratio followed by Ind. The distribution patterns for Pyr, Fla, Baa, Chr and Bkf appear to be slightly different from the Bbf and Ind, while very high concentrations were also recorded at the same sampling sites as Bap and Ant (Figure 2d).

The predominance of 3–5 ring compounds in the contaminated soils suggests the sources of PAHs might be ascribed to long time industrial activity and heavy traffic in these areas. Sun et al. reported that LMWPAHs (less than 4 rings, such as Nap, Ant, Phe, Flu, Acel and Ace) were mainly generated by low-or moderate-temperature combustion processes (such as biomass combustion and domestic coal burning), while HMWPAHs (ring number greater than 3) were mainly generated by high-temperature combustion process (such as vehicular exhausts and industrial coal combustion) [28]. In the sampling areas, there were very heavy traffic emissions (traffic, industrial, etc.). Thus, the larger fraction of HMWPAHs in the soils might stem from traffic exhausts and industrial coal combustion, meanwhile, whereas the soils sampled from AS had lower ∑16PAHs was lower than others areas, but higher ratio of HMWPAHs (78.7%) to the ∑16PAHs and 6-ring PAHs (14.5%) than others areas. 

Due to their physic-chemical proportions, such as higher biodegradability, solubility, volatility and lower sorption ability to soil, LMWPAHs levels accumulated in soil were lower than heavier ones. The presence of 2-ring and 3-ring PAHs in the surface soils can suggest that the more PAHs come from the recent deposition. Moreover, as the far more away from the pollution sources for AS than others, so the ∑16PAHs was lower. It is known that the crops can degrade some of PAHs, and LMWPAHs have higher biodegradability properties than the HMWPAHs [15,16], so in the soils sampled from AS have higher ratio of HMWPAHs (78.7%) to the ∑16PAHs and 6-ring PAHs (14.5%) than others areas. We will study these facts further.

### 3.3. Characteristics of PAHs Concentration in Vertical Distribution

**Figure 3:**
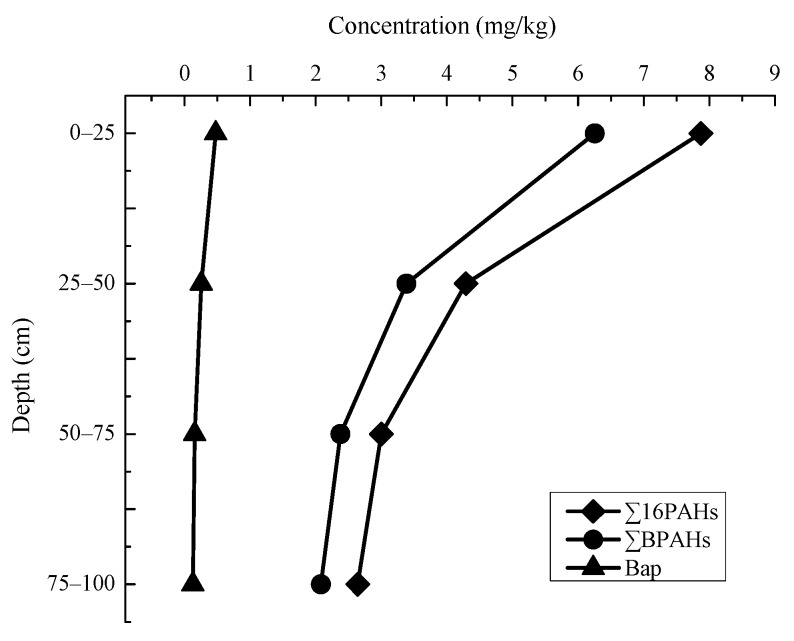
Vertical distribution profile of the total PAHs (∑16PAHs), total carcinogenic PAHs (∑BPAHs) and Bap in the soil profiles of samples around the chemical plant area.

Vertical distribution of PAHs in contaminated soils was assessed from the soil samples collected from vertical sections at four depths in the sampling areas. Figure 3 shows a representative vertical distribution profile of ∑16PAHs, ∑BPAHs and Bap in the area. It is expected that ∑16PAHs would gradually decrease with the increasing depth, from the top layer (0–25 cm) to bottom layer (75–100 cm) at each sample site, resulting in decreasing ∑16PAHs from 7.87 mg/kg to 2.64 mg/kg. Compared to the top layer soils the ∑16PAHs in 75–100 cm depth decreased by 66.5% in the sample area. This indicates that the PAHs probably came from pyrolysis inputs due to industrial emissions in the industrial activities and also shows the migrate trend of PAHs in the vertical section of the soils.

Moreover, the distribution profiles of ∑BPAHs and Bap in different vertical sections are shown on Figure 3. As expected that top layer soils contain the highest ∑BPAHs and Bap with 6.25 mg/kg and 4.76 mg/kg, respectively, and gradually decreased in the flowing lower vertical sections: 3.38 mg/kg and 2.98 mg/kg in 25–50 cm, 2.38 mg/kg, 1.55 mg/kg in 50–75 cm and 2.08 mg/kg, 0.128 mg/kg in 75–100 cm.

The study data suggest that Bap threshold value of 0.030 mg/kg applied in Polish regulations [29], 0.10 mg/kg suggested in Denmark [30] and Canada [31], and 0.50 mg/kg in Chinese GB 15618-2008 [32] be too strict as compared to the Bap limit value. If the 0.10 mg/kg value is applied, then the Bap concentration in the sample sites around the chemical plant is 100% higher than the threshold value. The worldwide regulatory guidance values for PAHs were usually derived from exposure and human health risk model (cancer risk or non-cancer risk), ecological risk assessment, or occupational [33]. If the Bap concentration in the sample sites around the chemical plant is 100% higher than the threshold value, it means that there would be great health and environmental risk concerns. So I think that is the reason why this study should be valued.

### 3.4. Characteristics of PAHs Component in Vertical Distribution

**Figure 4:**
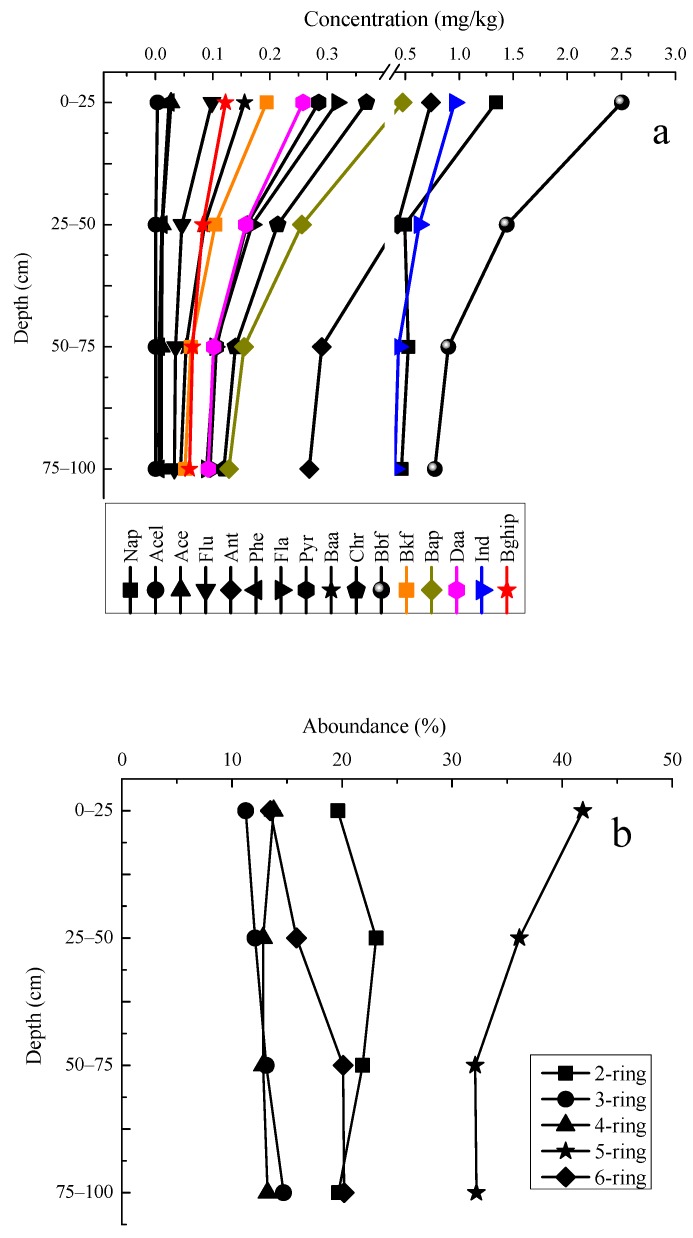
Concentration distributions in the vertical sections of (**a**) individual PAHs and (**b**) individual PAHs grouped by ring numbers in the soil profiles around the chemical plant area. Note: 2-ring: Nap; 3-ring: Ace, Acel, Flu, Phe, Ant; 4-ring: Fla, Pyr, Baa, Chr; 5-ring: Bbf, Bkf, Bap, Daa; 6-ring: Ind, Bghip.

Figure 4a presents individual PAHs content profiles in different vertical sections. Bbf, Ind, Ant and Nap were determined as the four main PAHs in the sample soils. The vertical distribution characteristics of individual PAHs (except Nap) appeared to be nearly the same. The highest concentration were obtained in the 0–25 cm top layers, and decreased with depth. 

The contents of individual PAHs as grouped by ring numbers in the profiles follows an order of 5-ring > 2-ring > 6-ring > 4-ring > 3-ring. 5-ring PAHs was predominant and varied from 32.1 to 41.9%, followed by 2-ring PAHs ranged from 19.6 to 23.1% (average 16.9%) and 6-ring PAHs determined in a range of 13.5–20.2% (average 13.9%) (Figure 4b). The 3-ring and 4-ring PAHs appeared to be less distributed in the top layer and the contents remained stable along the vertical sections. The reason for this is that the 3 and 4-ring PAHs were the major pollutant types in the areas, but they are relatively easily degraded by microbes in surface soils. The variation of 5-ring PAHs, as a dominant one in the soil profile, was more significant than other PAHs, the reason being the 5-ring PAHs were the most pollutant types and have extensive sources in the areas. It is interesting to note that 6-ring PAHs increased within the vertical sections, the reason assumed to be their higher hydrophobicity and molecular weight, the movement speed into deeper soil and the increasing capability to withstand biodegradability in the soil. Because of the high volatility, the relative abundance of 2-ring PAH was lower in the top and bottom soils, but higher in the middle soils [11,22].

### 3.5. Identification of PAH Sources

#### 3.5.1. Diagnostic Ratio

Diagnostic ratio is a widely used technique to apportion the origin and sources of PAHs present in different environmental media [14,34]. Although the use of diagnostic ratios has known uncertainties [35,36], many researchers have used this method as a useful approach to determine the sources of PAH sat special locations, such as areas close to the point sources, and the soil around industrial districts [37]. Therefore diagnostic ratio was used in this study as an indicative way to give insight about the PAH sources. In the reported studies, the diagnostic ratio was determined for different PAHs of interest, as summarized in Table 2.

According to a previous report, the ratio of Ant/(Ant + Phe) < 0.1 is taken as an indication of petroleum while a ratio >0.1 indicates a dominance of combustion [14]. Yunker suggested that a Fla/(Fla + Pyr) ratio <0.4 indicates petroleum input, ratio between 0.4–0.5 liquid fossil fuel (vehicle and crude oil) combustion and ratio >0.5 coal, wood or grass combustion source, and a Baa/(Baa + Chr) ratio of <0.2 usually implies a petroleum source, a ratio between 0.2–0.35 indicates either a petroleum or a combustion source, and a ratio of >0.35 denotes combustion source [34]. According to the literature [38], the value of Ind/(Ind + Bghip) >0.5 indicates grass/coal/wood combustion sources, a ratios between 0.20–0.50 implies fuel combustion sources (vehicle and crude oil), and ratios <0.20 indicate petrogenic origin. The ratio Bap/Bghip >0.6 indicates traffic sources and a Bap/(Bap + Chr) ratio of <0.2 indicates a petroleum source, a ratio between 0.2–0.35 indicates coal, wood or grass combustion source, and a ratio of >0.35 denotes vehicular combustion source [39,40]. The ratio of specific PAHs was determined to identify potential sources. Usually, the value of high molecular weight (HMW)/low molecular weight (LMW) PAHs < 1 implies mainly derived from petrogenic source and HMW/LMW PAHs > 1 implies mainly derived from pyrogenic source [41,42]. Phe/Ant ratio < 10 and Fla/Pyr ratio > 1 indicate that PAHs come from pyrogenic source, and Phe/Ant > 15 and Fla/Pyr < 1 indicate petrogenic origins of PAHs [40].

**Figure 5:**
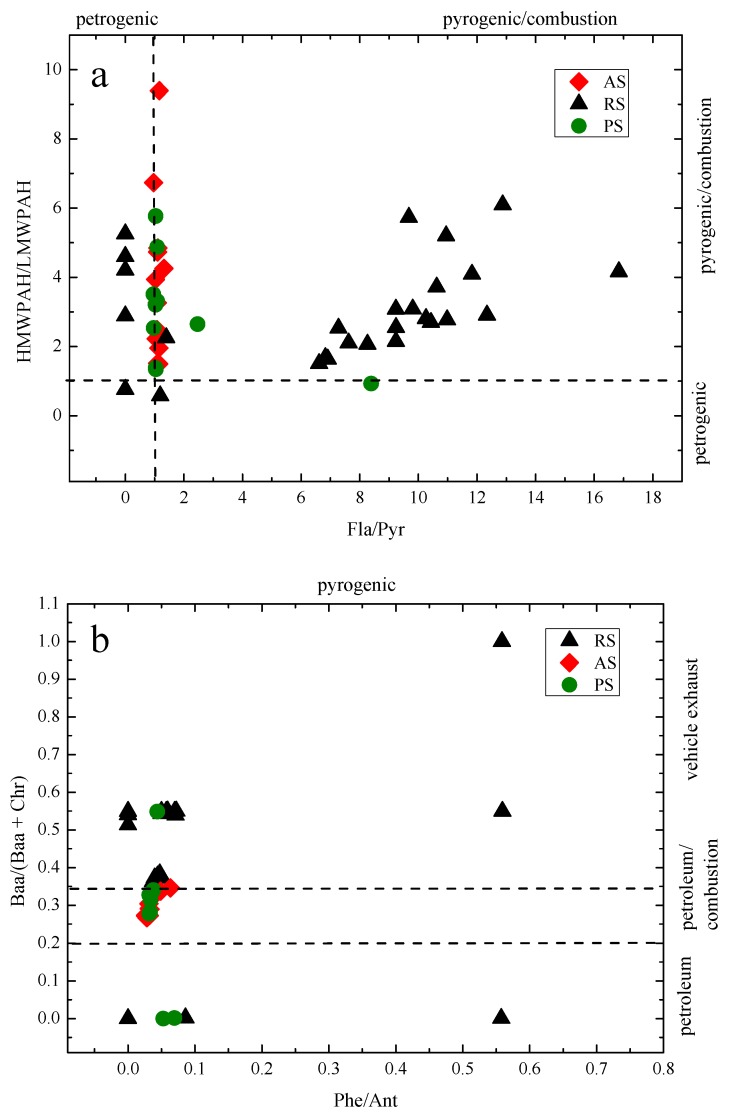

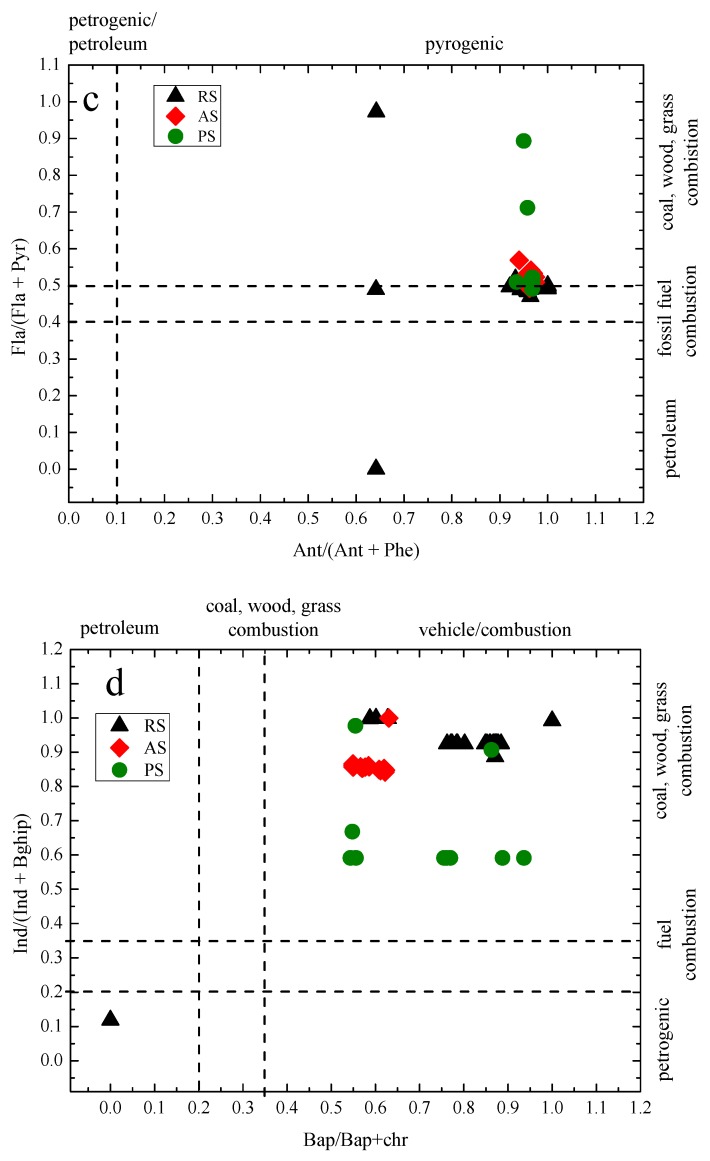
Diagnostic ratio charts for (**a**) HMW/LMW and Fla/Pyr, (**b**) Baa/(Baa + Chr) and Phe/Ant, (**c**) Fla/(Fla + Pyr) and Ant/(Phe + Ant), and (**d**) Bap/(Bap + Chr) and Ind/(Ind + Bghip) in the sample soils around the chemical plant.

Our data in Figure 5 shows that the ratios of Ant/(Ant + Phe) varied from 0.64 to 1.00, indicating that pyrogenic activities were the predominant PAH sources. The plot of HMWPAH/LMWPAH against Fla/Pyr shows that 95.5% of HMWPAH/LMWPAH > 1 and 87.9% of Fla/Pyr > 1, indicating predominantly pyrogenic sources. Figure 5b shows that the representative diagnostic ratios were Phe/Ant 0–0.56, Baa/(Baa + Chr) 0–1.00, 33.3% of Baa/(Baa + Chr) 0.2–0.35, and 60.6% of Baa/(Baa + Chr) > 0.35. These characteristic ratios indicate that the PAHs in the studied areas were predominantly pyrogenic, coal/wood/grass/vehicular combustion mixed sources. The plot of Fla/(Fla + Pyr) against Ant/(Ant + Phe) shows that the ratio of Fla/(Fla + Pyr) from 0 to 0.89, and 75.8% of Fla/(Fla + Pyr) > 0.5, 22.7% of Fla/(Fla + Pyr) ranged 0.4–0.5, and Ant/(Ant + Phe) < 10 in the sampling sites (Figure 5c). These results reveal that the PAHs originated from coal/wood/grass/petroleum combustion. Figure 5d shows that the 98.5% surface sample soils had a ratios of Bap/(Bap + Chr) > 0.35, indicating a vehicular combustion source, and a ratio of Ind/(Ind + Chr) ranged from 0.59 to 1.0, which were predominantly sourced from coal/wood/rass and vehicular combustions.

#### 3.5.2. Principal Component Analysis

**Figure 6:**
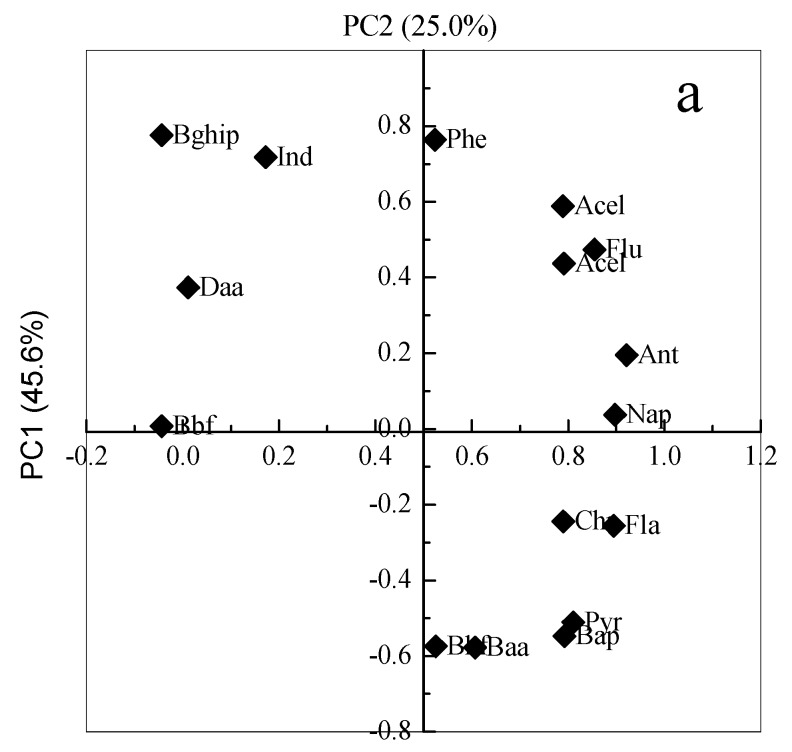

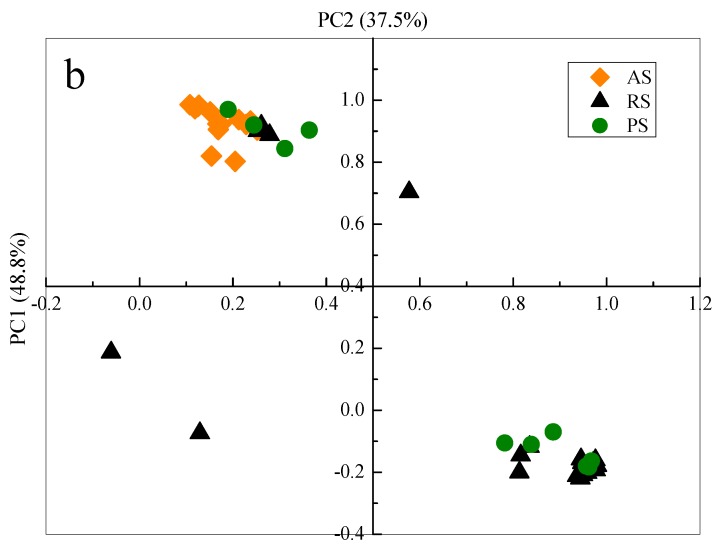
Principal Component Analysis loading plot for (**a**) 16 individual PAHs and (**b**) different sample sites.

Principal Component Analysis (PCA) is applied to extract valuable information from multivariate. PCA can also be used to analyze the sources of PAHs [44,45]. Each principle component (PC) is extracted with different factor loadings and further evaluated and recognized by source markers or profiles as reasonable pollution sources. To obtain more insight into the data characteristic, PCA was performed with individual PAH contents as active variables and all the sample sites as subjects (Figure 6a).

The five components were identified by the PCA from our data. The majority of the variance (86.3%) was explained by two eigenvectors factors: PC1 and PC2. PC1 was responsible for 48.8% of variance, and was mainly loaded from Ant (|r| = 0.922), Nap (|r| = 0.897), Fla (|r| = 0.895), Flu (|r| = 0.854), Pyr (|r| = 0.811), and slightly less located by Bap (|r| = 0.792), Chr (|r| = 0.790), Ace (|r| = 0.789), Baa (|r| = 0.607) and Phe (|r| = 0.523). The PC2 was explaining 37.5% of variance, was mainly dominated by Bghip (|r| = 0.776), Phe (|r| = 0.764) and Ind (|r| = 0.718), and followed by Acel (|r| = 0.589), Baa (|r| = 0.577), Bkf (|r| = 0.574) and Bap (|r| = 0.548) (Figure 6a). Figure 6aillustrates that the 16 PAH congeners were separated into three distinct groups on the factor loading plot. The first group included Nap, Baa, Fla, Pyr, Chr, Flu and Ind, which have been moderately loaded in PC1and PC2. The second group included one species of Bbf, which showed a higher loading value inPC2 than PC1. The third group included Bap, Bkf, Ant, Daa, Acel, Bghip, and Phe, which projected higher loading values inPC1 than PC2.

It is known that different types of emission sources result in typical PAH compositions [46,47]. Ace, Flu, Ant, Phe, Pyr, Bkf, Bap, and Fla are species associated with combustion (coal, wood, and diesel), incineration, and vehicle exhaust. Among them, Flu, Phe, Fla, Baa, Bkf, and Chr have generally been identified as originating from coal combustion [48], and Ant was recognized as a marker for wood combustion [49], Ace is usually used to trace grass or wood combustion [50]. Furthermore, Nap, Pyr, Acel, Bghip, Bbf, and Ind have been identified as indicators of vehicular emissions [51,52], among which Bbf as an indicators of diesel emission [53]. Pyr is an indicator of gasoline vehicle emissions. Nap, Flu and Phe were found in the vehicle emissions of gasoline-powered, diesel fuels and kerosene engines [54]. Moreover, Ace, Phe, Bkf, and Bap are also the predominant PAHs species derived from the sintering process [55], while, Nap, Ace, Flu, Phe, Ant and Fla are the major PAHs emitted from coke ovens [56,57]. Therefore, PAHs in the sample soils surrounding the chemical plant can be attributed to pyrogenic sources, including coal, wood, and coke combustion, which were the major PAHs responsible for the PC1. The gasoline-powered, diesel fuels and kerosene engines emissions were the major PAHs responsible for the PC2. The latter was less (37.5%) contributed to the pollution in the soils around the chemical plant areas.

PCA was performed with all the sample sites as active variables and concentration of 16PAHs in the individual sample soils as subjects (Figure 6b). These PAHs congeners are less vulnerable to different sites and four principal components that have been extracted collectively represent 87.4% of the data variance. The former two principal components collectively represent 70.5% of the variance, while the first principal component explains 45.6% of the variance, and the second principal component explains 25.0%. 

Referring to Figure 6b, the sample sites were separated into three relatively distinct groups on the factor-loading plot. High scores were observed with RS samples (group 1), which are located near the industrial district and high traffic volume areas. The lowest scores were observed in AS samples (group 2), which are located the areas far from the industrial and heavy traffic activities. Medium scores were mainly in the PS samples (group 3). The results show that the sources of PAHs for the three different function areas (AS, RS, and PS) are similar with less distinction.

## 4. Discussion

According to the European classification system of soil contamination [58], Σ16PAHs < 0.20 mg/kg show no contamination, 0.20–0.60 mg/kg corresponds to weak contamination, 0.60–1.0 mg/kg represents moderate contamination, and >1.0 mg/kg indicates heavy contamination, so all the sampling sites in this study were heavily contaminated, where by the PAHs concentrations are 12.6, 65.5 and 22.3 times higher, respectively, than the heavily contaminated level (1.0 mg/kg). It is indicated that the soil around the chemical plant stored great amount of PAHs and regulatory measures are needed to prevent the fields from turning into pollution sources, which would transfer PAHs into the air or groundwater in the region.

Due to their physic-chemical proportions, such as higher biodegradability, solubility, volatility and lower sorption ability to soil, LMWPAHs levels accumulated in soil were lower than heavier ones [59]. The presence of 2-ring and 3-ring PAHs in the surface soils can suggest that the more PAHs come from recent deposition events.

In general, the highest concentration of ∑PAHs and ∑BPAHs were observed in the surface section. The highest concentrations of some PAHs, such as Phe, Fla, Chr, Bbf, Bap, Baa and Bkf showed the same pattern. These results suggested that the long term production activities of the chemical plant had an obvious effect on the PAH distribution in soil vertical sections. The PAHs in soil could be ascribed to the most recent emission and long-time accumulation from the chemical plant’s long-term operation involving burning of coal and heavy transport activity. PAH distributions in soil vertical sections appeared to have received little attention in the reported studies. No report investigating the PAH distribution in deep soils contaminated by a chemical plant were found in the literature. Jin reported the vertical transport and distribution of PAHs in soils from the wastewater irrigation areas, indicating that LMW PAHs underwent a higher migration rate than the HMWPAHs along the vertical sections [60]. Our results from this study disagree with their conclusions. This can be explained by the fact that that the PAHs in our study area were mainly released from incomplete coal combustion in the chemical plant and the long-term accumulation. On the other hand, there was still a certain amount of PAHs that migrated downward and penetrated slowly into the deep soil sections because of factors like rainwater leaching and combination with soil clay.

The sources of PAHs in the soils around the chemical plant might be categorized into two distinctive groups. About 85.5% and 14.5% of the sampling sites exhibited the typical attributes of coal combustion and emissions of vehicular traffic, respectively. The contribution of PAHs in the areas was from coal/wood/grass combustion, and the traffic emissions, thus indicating that the surface soils from the areas around the chemical plant were strongly affected by its production activity.

According to the PCA of our data two typically components could be identified and believed to represent the following source categories: (1) combustion source (including coal, wood, and diesel combustion), (2) vehicular exhaust (including gasoline-powered, diesel fuels and kerosene engines), and the primary sources of PAHs in the AS, RS, and PS sample soils.

The above data demonstrate that there is a high ecological health risk in the environment around the chemical plant. The reason attributed to the long time industrial activity and the heavy traffic serving the plant in the areas. Zhang reported that the HMWPAHs reflect the existence of significant combustion products from low temperature pyrolytic processes and/or petrogenic sources [8]. Many previous studies have reported that during coal combustion PAH formation and emission mechanisms include two processes: pyrolysis and pyro-synthesis. In a pyrolysis process the coal partially is cracked into smaller and unstable fragments, mainly highly reactive free radicals with a very short average lifetime. In a pyro-synthesis process more stable PAHs (such as Bap and other HMWPAHs) are formed through recombination reactions [61,62,63,64]. 

## 5. Conclusions

The concentrations of the priority control 16 PAHs in the 66 soils samples around the chemical plant varied from 3.87 mg/kg to 116 mg/kg. Possibly carcinogenic PAHs ranged from 3.11 mg/kg to 94.2 mg/kg. The highest PAHs were distributed in the road sites. The most abundant PAHs were Bap (average 7.64 mg/kg), followed by Ant (4.62 mg/kg) and Bkf (4.23 mg/kg). It was notable that the concentration of Bap (Group 1 carcinogen) in the road site surface soils reached as high as 42.3 mg/kg.

The vertical distribution of ∑16PAHs, ∑BPAHs and Bap showed gradually decreased with the soil vertical sections, and the contents of ∑16PAHs, ∑BPAHs and Bap in topsoil (0–25 cm) were higher 2.98–37.8 times than in deep soil (75–100 cm) for the three sampling sites.

The results have shown that PAHs are present in high concentrations in the top soil of all the sample areas near the chemical industry. The sources of PAHs may mostly be ascribed to the processes of the chemical industries during handling and transportation of the raw materials and products. 90.3% of PAHs were mainly sourced from coal/wood/grass combustion processes, followed by the petroleum combustion processes (19.7%).

## Figures and Tables

**Table 1:** Concentrations of individual PAHs, ∑16PAHs and ∑BPAHs in the samples from agricultural soil (AS), roadside soil (RS) and parkgreen space soil (PS) around the chemical plant.

PAH Compound	Abbr.	Carcinogenicity Classification	No. of Ring	Agriculture Soils (AS, mg/kg)			Roadside Soils (RS, mg/kg)			Park Green Space Soils (PS, mg/kg)		
				Range	Mean	Median	Range	Mean	Median	Range	Mean	Median
Naphthalene	Nap	2B	**2**	0.007–4.87	1.51	1.5	1.15–8.93	5.17	4.7	0.337–10.6	2.72	1.61
Acenaphthylene	Acel		3	ND-0.022	0.006	0.003	0.11–0.240	0.064	0.044	ND-0.109	0.027	0.016
Acenaphthene	Ace		3	ND-0.103	0.032	0.025	0.056–1.79	0.412	0.263	0.015–0.517	0.126	0.08
Fluorene	Flu		3	0.015–0.399	0.104	0.077	0.405–7.86	2.14	1.64	0.053–2.62	0.615	0.41
Anthracene	Ant		3	0.332–4.26	0.992	0.701	3.62–23.0	9.45	8.36	0.485–13.1	3.69	2.84
Phenanthrene	Phe		3	0.009–0.168	0.038	0.024	ND-12.9	1.37	0.435	0.016–0.907	0.181	0.12
Fluoranthene	Fla		4	0.140–2.41	0.546	0.397	ND-10.3	4.97	4.59	0.058–6.53	1.63	1.11
Pyrene	Pyr		4	0.135–2.49	0.502	0.341	0.288–10.2	4.72	4.58	0.007–6.29	1.44	1.00
Benz [a] anthracene	Baa	B	4	0.071–1.71	0.288	0.149	ND-6.02	2.51	2.43	ND-1.32	0.533	0.403
Chrysene	Chr	2B	4	0.190–3.49	0.62	0.348	ND-7.96	3.54	3.74	0.216–3.52	1.31	1.11
Benzo [b] fluoranthene	Bbf	2B	5	1.36–25.5	4.44	2.52	ND-39.0	3.35	0.327	ND-6.36	1.53	0.153
Benzo [k] fluoranthene	Bkf	2B	5	0.100–9.90	0.985	0.229	ND-19.5	8.97	10.4	ND-6.01	2.45	1.73
Benzo [a] pyrene	Bap	B	5	0.232–5.95	0.973	0.49	ND-35.3	16.1	15.8	0.435–22.1	5.55	4.01
Dibenz [a,h] anthracene	Daa	B	5	0.150–3.10	0.514	0.265	0.010–2.61	0.276	0.01	0.010–0.299	0.045	0.01
Indeno[1,2,3-cd]pyrene	Ind	2B	6	0.566–11.6	1.83	0.927	0.087–39.5	2.34	0.087	0.087–2.07	0.469	0.087
Benzo [g,h,i] perylene	Bghip		6	0.007–0.343	0.163	0.142	0.007–0.642	0.043	0.007	0.007	0.007	0.007
∑16PAHs				3.87–76.0	12.6	7.55	35.4–116.	65.5	60.7	5.93–66.5	22.3	19.9
∑BPAHs				3.22–66.1	11.2	5.99	20.2–94.2	42.3	39.4	3.82–36.4	14.6	16.8

*Note*: ND represents not detected; B, a group of PAHs which are probably carcinogenic to human [2]; 2B which are possibly carcinogenic to human; All other PAHs compounds are currently regarded as ‘not classifiable’ [3].

**Table 2:** Diagnostic ratios reported for PAHs in soils.

PAHs	Diagnostic Ratio	Sources	Reference
**Ant/(Ant + Phe)**	<0.1	Petroleum	[14]
	>0.1	Combustion	
**Fla/(Fla + Pyr)**	<0.4	Petroleum	[34]
	0.4–0.5	Liquid fossil fuelcombustion	
	>0.5	Coal, wood or grass combustion	
**Baa/(Baa + Chr)**	<0.2	Petroleum	[37]
	0.2–0.35	Petroleum or combustion	
	>0.35	Combustion	
**Phe/Ant**	<10	Pyrogenic	[40]
	<15	Petrogenic	
**Ind/(Ind + Bghip)**	<0.2	Petrogenic	[38]
	0.2–0.5	Fuel combustion(vehicle and crude oil)	
	>0.5	Grass/coal/wood combustion	
**Bap/(Bap + Chr)**	<0.2	Petroleum	[39,40]
	0.2–0.35	Coal, wood or grass combustion	
	>0.35	Vehicular combustion	
**HMW/LMW PAHs**	>1	Pyrogenic	[41]
	<1	Petrogenic	[42]
**Fla/Pyr**	<1	Petrogenic	[43]
	>1	Pyrogenic	[39]
**Bap/Bghip**	>0.6	Traffic	[40]
